# Butyrate Suppresses Glucose Metabolism of Colorectal Cancer Cells *via* GPR109a-AKT Signaling Pathway and Enhances Chemotherapy

**DOI:** 10.3389/fmolb.2021.634874

**Published:** 2021-03-29

**Authors:** Hong-Wei Geng, Feng-Yi Yin, Zhi-Fa Zhang, Xu Gong, Yun Yang

**Affiliations:** Beijing Advanced Innovation Centre for Biomedical Engineering, Key Laboratory for Biomechanics and Mechanobiology of Ministry of Education, School of Biological Science and Medical Engineering, Beihang University, Beijing, China

**Keywords:** butyrate, colorectal cancer cell, glucose metabolism, glycolysis inhibition, chemotherapy

## Abstract

Glycolysis inhibitors are promising therapeutic drugs for tumor treatment, which target the uniquely elevated glucose metabolism of cancer cells. Butyrate is a critical product of beneficial microbes in the colon, which exerts extraordinary anti-cancer activities. In particular, butyrate shows biased inhibitory effects on the cell growth of cancerous colonocytes, whereas it is the major energy source for normal colonocytes. Besides its roles as the histone deacetylases (HDACs) inhibitor and the ligand for G-protein coupled receptor (GPR) 109a, the influence of butyrate on the glucose metabolism of cancerous colonocytes and the underlying molecular mechanism are not fully understood. Here, we show that butyrate markedly inhibited glucose transport and glycolysis of colorectal cancer cells, through reducing the abundance of membrane GLUT1 and cytoplasmic G6PD, which was regulated by the GPR109a-AKT signaling pathway. Moreover, butyrate significantly promoted the chemotherapeutical efficacy of 5-fluorouracil (5-FU) on cancerous colonocytes, with exacerbated impairment of DNA synthesis efficiency. Our findings provide useful information to better understand the molecular basis for the impact of butyrate on the glucose metabolism of colorectal cancer cells, which would promote the development of beneficial metabolites of gut microbiota as therapeutical or adjuvant anti-cancer drugs.

## Introduction

Inhibition of glycolysis in cancer cells is an emerging and powerful approach to combat cancer (Hay, 2016). Increased glucose uptake and enhanced glycolysis has been identified as a hallmark of cancer cells, with upregulated levels of transporters and enzymes involved in glucose metabolism (Liberti and Locasale, 2016). The accelerated glucose metabolism of cancer cells provides sufficient metabolite precursors and energy to sustain fast cell growth (Hanahan and Weinberg, 2011; Al-Shammari et al., 2019), and the dependence on such metabolic alteration of cancer cells provides a reliable target to specifically eliminate or suppress tumor growth by inhibition of glycolysis (Liu et al., 2020). Exploration of more glycolysis inhibitors as potential anticancer drugs is significant to promote the development of anticancer therapy, which is especially important during the global pandemic of coronavirus disease 2019 (COVID-19), since tumor management of immunotherapy and surgery was observed to increase the severity of infectious outcome of some patients with both COVID-19 and cancer (Finley et al., 2020).

Butyrate is a short-chain fatty acid (SCFA) produced by beneficial commensal bacteria in the colon, which exhibits extraordinary anti-cancer activities (Liu et al., 2018). A plenty of studies have been performed to elucidate the molecular mechanism for the anticancer effects of butyrate. Through inhibiting histone deacetylases (HDACs), butyrate could inactivate several oncogenic signaling pathways in cancerous cells (Wan et al., 2017), e.g., mitogen-activated protein kinase 1 (MAPK1) signaling pathway which inhibits apoptosis and promote rapid proliferation of cancer cells (Li et al., 2017), and small mothers against decapentaplegic homolog 3 (SMAD3) signaling pathway, one of the key activators in the process of epithelial-mesenchymal transition (Wawruszak et al., 2019). Butyrate has also been identified as a ligand for G protein-coupled receptor 109a (GPR109a) in cancerous cells, which regulates tumor growth by activating the downstream signal cascade of GPR109a (Thangaraju et al., 2009). The WNT signaling pathway, which is able to modulate the expression of various oncogenes, could be suppressed by the activation of GPR109a by butyrate, leading to the impairment of tumor growth (Chen et al., 2020). Notably, butyrate has been shown to preferentially inhibit the proliferation of cancerous colonocytes, whereas supply energy source for normal colonocytes (Liu et al., 2018). Besides revealing its role in activating the GPR109a mediated signaling pathway and acting as the HDACs inhibitor (Koh et al., 2016), its influence on the glucose metabolism of cancerous colonocytes and the underlying molecular mechanism are not fully explored.

In this work, the impact of butyrate on the glucose transport and glycolysis activity of colorectal cancer cells was investigated, and the signal transduction process mediating such effects was explored. It was found that butyrate significantly suppressed the glucose metabolism of cancerous colonocytes by reducing the abundance of membrane GLUT1 and cytoplasmic G6PD, which was mediated by the GPR109a-AKT signaling pathway. Moreover, butyrate markedly promoted the chemotherapeutical efficacy of 5-fluorouracil (5-FU) on colorectal cancer cell, by further inhibiting the DNA synthesis efficiency with 5-FU. Our results provide useful information of the suppressing effects of butyrate on the glucose metabolism of colorectal cancer cells as well as the underlying molecular mechanism, which would promote the development of butyrate as a therapeutical or adjuvant anti-cancer drug.

## Materials and Methods

### Cell Lines and Cell Culture

The Human colorectal cancer cell lines, HCT116 and LoVo cells, were obtained from Cell Bank, Type Culture Collection, Chinese Academy of Science. HCT116 cells were grown in McCoy’s 5A medium (Sigma-Aldrich, United States), and LoVo cells were cultured in DMEM/F12 medium (DMEM medium and F12 medium were mixed with ratio of 1:1, Gibco, United States). Both cell lines were maintained in a humidified incubator with 5% CO_2_ at 37°C, supplemented with 10% fetal bovine serum (Gibco, United States) and 1% penicillin-streptomycin.

### RNA Interference

The GPR109a targeting siRNA and control siRNA were synthesized by Genepharma (Jiangsu, China). The siRNAs were delivered into HCT116 and LoVo cells using lipofectamineTM RNAiMAX reagent (Invitrogen, United States) in an antibiotic-free culture medium, by following the manufacturer’s instructions. The sequences of siRNAs applied in this study were described in Supplementary Table S1.

### Western Blotting

The cells were collected and washed twice with ice-cold PBS, and were subsequently lyzed in RIPA buffer (50 mM Tris pH 8, 150 mM NaCl, 1% NP-40, 0.5% deoxycholic acid, 274 and 0.1% SDS) supplemented with protease and phosphatase inhibitors (1 mM PMSF, 5 μg/ml 275 leupeptin, 2 μg/ml aprotinin, 1 mM EDTA, 10 mM NaF, and 1 mM NaVO_4_). The lysates were centrifuged for 10 min at 10,000 *g* at 4°C. The supernatants were collected, and the protein concentration was determined using Bradford assay kit (Beyotime, China). The protein was separated on 12% SDS-PAGE, which was then transferred to polyvinylidene difluoride (PVDF) membranes (Millipore, United States). The PVDF membranes were blocked by 5% nonfat milk, and was incubated with the indicated primary-antibody solution at 4°C overnight, followed by incubation with peroxidase-conjugated secondary antibodies for 1.5 h. The resulting bands were tested using chemiluminescent reagents on a ChemiDoc XRS system (Bio-Rad, United States). Antibodies against *β*-actin (1:2000), AKT (1:1000) and AKT (phospho-Ser473, 1:1000) were purchased from Bioworld (China). Antibodies against GLUT1 (1:1000) and G6PD (1:1000) were purchased from Cell Signaling Technology (United States).

### Measurements of Central Metabolites Associated in Glucose Metabolism

To measure the levels of intracellular metabolites, cell extracts were prepared from 2 × 10^6^ to 6 × 10^6^ HCT116 cells with methanol containing Internal Standard Solution (Human Metabolime Technoligies), which was analyzed using a capillary electrophoresis (CE)-connected ESI-TOFMS system. Briefly, a total of 2 × 10^6^ to 6 × 10^6^ cells in a 10 cm plate were washed twice with 5% mannitol, and was lyzed in 1.3 ml methanol containing 10 μM Internal Standard Solution. A 1.0 ml aliquot was mixed vigorously with 0.4 ml Milli-Q water and 1.0 ml chloroform, and was centrifuged at 2,300 *g* for 5 min at 4°C. The aqueous layer was filtered to remove proteins through a Millipore 5-kDa cutoff filter. The filtrate was lyophilized, resuspended in 50 µl Milli-Q water and analyzed using CE-TOFMS (Human Metabolome Technologies).

### Cell Viability Assay

HCT116 and LOVO cells were seeded with an initial density of 5 × 10^3^ cells/well in 96-well plates, and were cultured for 24 h before treatment. Cells were then treated by various testing reagents as described in the main context, and were incubated for 24 h before evaluation. Then, the cell culture medium was added with Cell Counting Kit-8 (Dojindo Molecular Technologies, Japan.), and was incubated at 37°C in the dark for 1 h. Absorbance at 590 nm was recorded using a microplate reader (BioTEK, United States).

### 5-Bromo-2′-Deoxyuridine (BrdU) Incorporation Assay

BrdU incorporation during DNA synthesis was evaluated by ELISA at 24 h after transfection with the BrdU kit (Beyotime Institute of Biotechnology, China), by following the manufacturer’s protocol. The experiment was implemented in triplicate, and the absorbance at a wavelength of 450 nm was recorded by a microplate reader (BioTEK, United States).

### Quantitative Real-Time Reverse Transcription Polymerase Chain Reaction Analysis

Total RNA extraction from HCT116 and LOVO cells was performed with TRIzol reagent (Invitrogen, United States). The concentration and quality of the extracted RNA were determined by a NanoDrop spectrophotometer (Thermo Scientific, United States). 1 μg RNA from each sample was reversely transcribed to cDNA with a PrimeScript^™^ IV 1st strand cDNA Synthesis Mix (Takara, China) according to the manufacturer’s instructions. The primer pairs for quantitative RT-PCR assay were described in Supplementary Table S2. Data were analyzed using the comparative threshold cycle (CT) method with *β*-actin serving as the internal control, and the fold change was calculated using the 2^−ΔΔCT^ method with a 7900HT Real-Time PCR system and software (Applied Biosystems, United States).

### Flow Cytometry

HCT116 and LoVo cells were incubated with a serum-free DMEM medium with low glucose levels for 24 h. Then HCT116 and LoVo cells were incubated with low glucose DMEM medium containing 100 μM 2-NBDG at 37°C for 30 min. 2-NBDG fluorescence was measured by flow cytometry (BD Biosciences, San Jose, CA, United States).

Cell apoptosis was assayed using FITC Annexin V Apoptosis Detection Kit (BD Pharmingen) according to the manufacturer’s instructions. HCT116 and LOVO cells were washed twice using ice-cold PBS, and resuspended with a concentration of 1 × 10^6^ cells/ml. 100 μl of cell suspension was supplemented with 5 μl FITC Annexin V and 5 μl propidium iodide (PI), and the resultant solution was incubated in the dark at room temperature for 15 min. Then cells were analyzed by flow cytometer (BD AccuriTM C6).

### Statistical Analysis

All data were obtained from independent triplicates, and were analyzed using Prism 5.0 software (GraphPad Software, United States). The results were subjected to one-way analysis of variance (ANOVA) followed by a Student-Newman-Keuls (SNK) test to assess the significance between different groups. *p* < 0.05 was considered statistically significant.

## Results

### Butyrate Inhibited Glucose Uptake and Membrane Content of GLUT1 in Colon Cancer Cells

**FIGURE 1:**
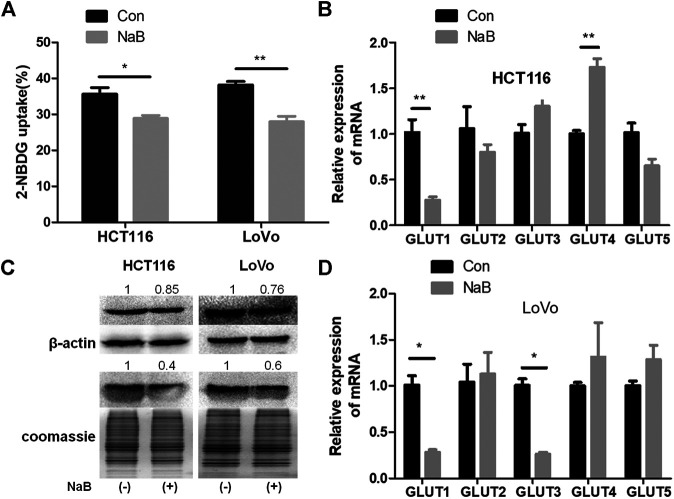
Butyrate inhibited glucose uptake and the membrane abundance of GLUT1 in colorectal cancer cells. **(A)** The glucose uptake efficiencies of HCT116 and LoVo cells being treated with 2 mM butyrate (annotated as “NaB”) or PBS vehicle (marked as “Con”) for 24 h were measured, using a fluorescent glucose analogue, 2-NBDG. **(B)** The mRNA levels of GLUT1-5 in HCT116 cells after incubating with 2 mM butyrate or PBS for 24 h were tested using quantitative PCR (qPCR). **(C)** The total protein level of GLUT1 (labeled as “GLUT1_t_”) and GLUT1 abundance in the membrane fraction (labeled as “GLUT1_m_”) in HCT116 and LoVo cells w/o butyrate treatment was measured using western blotting. Cell membrane proteins were extracted from both cell lines after addition of 2 mM butyrate or PBS for 24 h, and the abundance of membrane GLUT1 was measured by western blotting. Coomassie bright blue staining of polyacrylamide gels containing cell membrane proteins represented the concentration of cell membrane proteins. **(D)** The mRNA levels of GLUT1-5 were tested using qPCR in LoVo cells being incubated with 2 mM butyrate or PBS for 24 h. The error bars showed standard deviations from independent triplicates. *p < 0.05, **p < 0.01.

Glucose uptake is a vital target for inhibiting glucose consumption of cancer cells. To investigate the influence of butyrate on glucose transport in colorectal cancer cells, glucose uptake measurement using a fluorescent analogue of glucose molecule, 2-NBDG, was conducted in HCT116 and LoVo cell lines. After being treated with PBS vehicle or 2 mM butyrate for 24 h, the fluorescence of cells was measured by flow cytometry. In comparison with PBS buffer, butyrate treatment led to substantial decrease in the ratio of cells with absorbed fluorescent 2-NBDG, being dropped from 38 to 27% and from 36 to 24% in HCT116 and LoVo cells, respectively (Figure 1A). These results showed that butyrate significantly inhibited glucose uptake in colorectal cancer cell lines.

Glucose transporter 1-5 (GLUT1-5) have been reported to mediate glucose intake in various cell types and tissues (Mueckler and Thorens, 2013). In order to identify the specific glucose transporter sensitive to butyrate in colorectal cancer cells, expression levels of GLUT1-5 in HCT116 and LoVo cell lines being treated with PBS or butyrate for 24 h were compared. In the two colorectal cancer cell lines, around 70% decrease in GLUT1 expression resulting from butyrate addition was consistently observed, indicating a prominent role of butyrate in inhibiting glucose metabolism of colorectal cancer cells by reducing the expression level of GLUT1 (Figures 1B,D). And butyrate also regulates the mRNA level of GLUT1 in HT29 and HCT8 cells, suggesting that the regulation of GLUT1 in colorectal cancer cells is a common phenomenon (Supplementary Figure S1). The impact of butyrate on the abundance and distribution of GLUT1 at the protein level was further studied by measuring the total and membrane protein amount of GLUT1 (Figure 1C). The total content of GLUT1 in HCT116 and LoVo cells was significantly reduced by 15 and 24%, while the membrane fraction in HCT116 and LoVo cells was significantly reduced by 60 and 40% in response to butyrate treatment, respectively (Figure 1C). These results indicate that butyrate significantly reduces the expression level and membrane allocation of GLUT1, and consequently inhibits glucose transport in colorectal cancer cells.

### AKT Signaling Pathway Mediated the Impact of Butyrate on GLUT1

**FIGURE 2:**
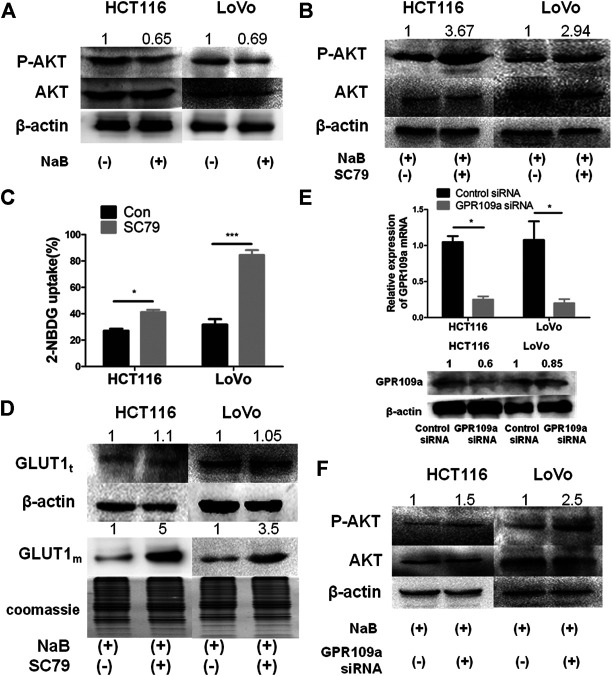
The inhibitory effect of butyrate on the glucose uptake of cancerous colonocytes was mediated by the AKT pathway. **(A)** The protein levels of total AKT and phosphorylated AKT (labeled as “P-AKT”) were tested by western blotting in HCT116 and LoVo cells after incubating with 2 mM butyrate (marked as “NaB”) or PBS vehicle (marked as “Con”) for 24 h. **(B)** When HCT116 and LoVo cells were co-incubated with 2 mM butyrate and 10.96 μM SC79 or incubated with 2 mM butyrate alone for 24 h, western blotting was applied to measure the protein levels of total AKT and phosphorylated AKT. **(C)** 2-NBDG, an indicator of glucose uptake efficiencies was measured using flow cytometry in HCT116 and LoVo cells after incubating with 2 mM butyrate or PBS vehicle for 24 h. **(D)** The protein levels of GLUT1 in whole cells (labeled as “GLUT1_t_”) or cell membrane (labeled as “GLUT1_m_”) were tested using western blotting, after HCT116 and LoVo cells were incubated with 2 mM butyrate alone or the combination of 2 mM butyrate and 10.96 μM SC79 for 24 h. Coomassie bright blue staining of polyacrylamide gels represented the concentration of cell membrane proteins. **(E)** The mRNA and protein levels of GPR109a were tested by qPCR and western blotting, respectively, at 24 h after the transfection of GPR109a-targeting siRNA or control siRNA into HCT116 and LoVo cells along with the addition of 2 mM butyrate. **(F)** The protein levels of total AKT and phosphorylated AKT were tested using western blotting in HCT116 and LoVo cells being incubated with 2 mM butyrate and transfected with GPR109a specific siRNA or control siRNA for 24 h. The error bars showed standard deviations from independent triplicates. *p < 0.05, ***p < 0.001.

AKT pathway was reported to play an important role in regulating the expression and membrane trafficking of GLUT1 (Zhao and Zhang, 2016). To investigate the underlying mechanism of butyrate in reducing the membrane abundance of GLUT1 and consequently decreasing glucose uptake, the impact of butyrate on the AKT signaling pathway was explored. After incubation with 2 mM butyrate or PBS for 24 h, the total protein amount of AKT in HCT116 and LoVo cells was found to be constant w/o butyrate treatment, while the phosphorylation level of AKT was significantly decreased by butyrate in both cell lines (Figure 2A). To verify whether butyrate exerts its impact on membrane content of GLUT1 through the AKT signaling pathway, SC79, an AKT phosphorylation activator, was added to the cells treated by butyrate to promote the expression of phosphorylated AKT (marked as “P-AKT”) and uptake of 2-NBDG (Supplementary Figures S2, S3). When co-incubated with 2 mM butyrate and 10.96 μM SC79 for 24 h, the suppressing effect of butyrate on phosphorylation level of AKT was restored by SC79 (Figure 2B). Moreover, compared with cells treated with butyrate alone, co-incubation with butyrate and SC79 led to an increased proportion of cells containing fluorescent 2-NBDG from 24 to 44% and from 39 to 80% in HCT116 and LoVo cells, respectively (Figure 2C). Notably, in comparison to cells treated with only butyrate, treatment with both butyrate and SC79 increased the membrane content of GLUT1 by five times and 3.5 times in HCT116 and LoVo cells, respectively (Figure 2D). These results demonstrated that the inhibitory effect of butyrate on the membrane content of GLUT1 and glucose uptake efficiency in colorectal cancers cells was restored by an AKT phosphorylation activating bioreagent, indicating that AKT pathway is essential for butyrate to inhibit glucose uptake of colorectal cancer cells.

GPR109a has been recognized as the primary membrane receptor for butyrate by many studies (Thangaraju et al., 2009), and the involvement of GPR109a in the AKT pathway mediated suppression on glucose uptake by butyrate was further investigated. After applying a GPR109a-targeting siRNA to HCT116 and LoVo cells, the expression levels of GPR109a in both cells were dramatically knockdown by around 80% (Figure 2E). In comparison to HCT116 and LoVo cells with butyrate treatment, both cell lines interfered with GPR109a-targeting siRNA along with butyrate addition showed an increase in the AKT phosphorylation level by about 1.5 and 2.5 times, respectively (Figure 2F). According to these results, the suppressing impact of butyrate on glucose uptake in colorectal cancer cells is mostly likely activated by GPR109a, which elicits a decreased level of phosphorylated AKT and subsequently reduced membrane content of GLUT1.

### Butyrate Inhibited G6PD Expression and DNA Synthesis in Colon Cancer Cells

**FIGURE 3:**
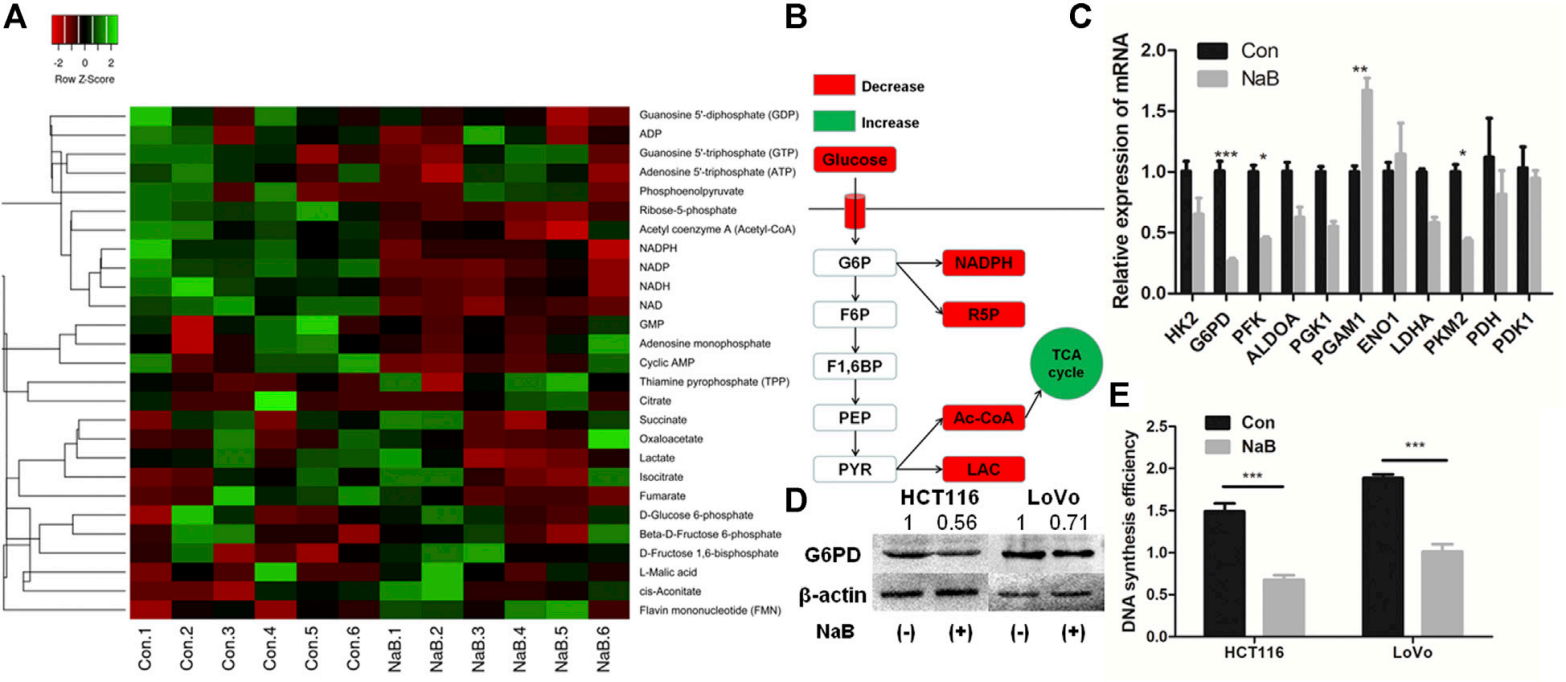
Butyrate suppressed G6PD abundance and DNA synthesis of colon cancer cells. **(A)** The concentration of intracellular metabolites associated with glucose metabolism in HCT116 cells being incubated with 2 mM butyrate (labeled as “NaB”) or PBS vehicle (marked as “Con”) for 24 h were measured by liquid chromatograph-mass spectrometer/mass spectrometer (LC-MS/MS). **(B)** The variation of central metabolites in HCT116 cells being incubated with 2 mM butyrate or PBS vehicle for 24 h was annotated in the schematic diagram of glycolysis pathway. **(C)** After HCT116 cells were incubated with 2 mM butyrate for 24 h, the expression levels of genes that regulate glucose metabolism were analyzed by qPCR. (**D)** The protein levels of G6PD in HCT116 and LoVo cells after being treated with 2 mM butyrate for 24 h were tested by western blotting. **(E)** After incubating HCT116 and LoVo cells with 2 mM butyrate or PBS vehicle for 24 h, DNA synthesis efficiency in both cell lines was measured by BrdU incorporation assay. The error bars showed standard deviations from independent triplicates. *p < 0.05, **p < 0.01, ***p < 0.001. G6P, D-Glucose 6-phosphate; F6P, Beta-D-Fructose 6-phosphate; F1,6BP, D-Fructose 1,6-bisphosphate; PEP, Phosphoenolpyruvate; PYR, Pyruvate; LAC, Lactate; R5P, Ribose-5-phosphate.

Besides the influence on the glucose uptake efficiency, the impact of butyrate on the glucose metabolism of colorectal cancer cells was further studied. The metabolites associated with glucose metabolism in HCT116 cells being incubated with 2 mM butyrate or PBS vehicle for 24 h were exacted and measured (Figures 3A,B). The content of lactate was reduced by 60% in HCT116 cells in response to butyrate treatment. As one of the main products of glycolysis in tumor cells, the reduction in lactate concentration indicates that glycolysis was suppressed by butyrate in HCT116 cells. Similarly, butyrate reduced the levels of ribose-5-phosphate (R5P), acetyl-CoA and NADPH in HCT116 cells by 70, 58, and 60%, respectively (Supplementary Figure S4).

The involvement of glucose metabolizing enzymes was further studied by analyzing their expressing levels in HCT116 cells treated with 2 mM butyrate or PBS for 24 h. Among the 11 genes related to glycolysis, the mRNA levels of glucose-6-phosphate dehydrogenase (G6PD), phosphofructokinase (PFK) and pyruvate kinase M2 (PKM2) and phosphoglycerate mutase 1 (PGAM1) in HCT116 cells were significantly influenced by butyrate treatment (Figure 3C). In particular, G6PD showed the most prominent fold change in expressing level, which was downregulated by 70% in response to butyrate. Consistently, the protein abundance of G6PD in both HCT116 and LoVo cell lines treated with butyrate for 24 h was decreased by 50 and 35%, respectively, compared with those in cells added with PBS vehicle for 24 h (Figure 3D).

G6PD is a key enzyme of the pentose phosphate pathway (PPP), which produces R5P for *de novo* synthesis of nucleotides (Ho et al., 2007). Based on the results that G6PD protein and R5P level were both dramatically downregulated by butyrate, we speculated that the DNA synthesis may be inhibited by butyrate treatment. In order to test such hypothesis, DNA synthesis efficiency of colorectal cancer cells after inoculation with 2 mM butyrate or PBS were probed by BrdU incorporation assay. It was showed that the DNA synthesis activities in both HCT116 and LoVo cells were remarkably reduced, indicated by the decrease in the optical density (OD) value of BrdU incorporation assay from 1.5 to 0.65 and from 1.83 to 0.97 in response to butyrate treatment, respectively (Figure 3E).

### Butyrate Regulated G6PD Expression and DNA Synthesis of Cancerous Colonocytes Through the AKT Pathway

**FIGURE 4:**
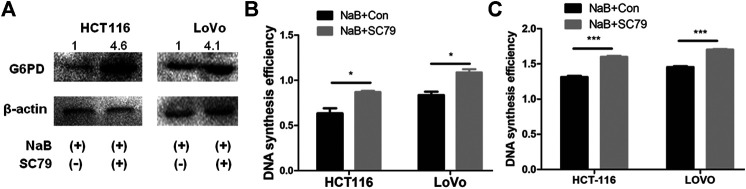
Butyrate regulated G6PD abundance and DNA synthesis of colon cancer cells through the AKT pathway. **(A**) The protein levels of G6PD were measured by western blotting in HCT116 and LoVo cells being treated with 2 mM butyrate alone or co-incubated with 2 mM butyrate and 10.96 μM SC79 for 24 h. **(B)** The effects of 10.96 μM SC79 (marked as “NaB + SC79”) or PBS vehicle (labeled as “NaB + Con”) on cell proliferation were tested by CCK-8 assay in HCT116 and LoVo cells which were incubated with 2 mM butyrate for 24 h. **(C)** After being treated with a combination of 2 mM butyrate and PBS vehicle (labeled as “NaB + Con”) or 2 mM butyrate and 10.96 μM SC79 (marked as “NaB + SC79”) for 24 h, the DNA synthesis efficiency in HCT116 and LoVo cells was assayed by BrdU incorporation experiment. The error bars showed standard deviations from independent triplicates. *p < 0.05, ***p < 0.001.

To further investigate how butyrate regulates G6PD abundance and DNA synthesis in colorectal cancer cells, the effects of AKT phosphorylation in such process were analyzed, which was proved to be inhibited by butyrate and mediated the suppression of glucose uptake in our above results. In comparison to HCT116 and LoVo cells added with 2 mM butyrate alone for 24 h, both cell lines incubated with 10.96 μM SC79 and 2 mM butyrate exhibited increased G6PD protein amount by 4.5 and 2.5 times, respectively (Figure 4A). SC79 also accelerated cell proliferation of HCT116 and LoVo cells with butyrate treatment in CCK-8 assay, with the OD value increased from 0.6 to 0.82 and from 0.8 to 1.1, respectively (Figure 4B). Furthermore, DNA synthesis efficiencies of HCT116 and LoVo cells being treated by 2 mM butyrate were promoted by SC79, indicated by the increase of OD value in BrdU incorporation assay from 1.37 to 1.64 and from 1.41 to 1.67, respectively (Figure 4C). These results indicated that AKT signaling pathway was closely involved in regulating G6PD content and DNA synthesis efficiency as well as membrane abundance of GLUT1 and glucose uptake rate of colorectal cancer cells.

### Butyrate Enhanced the Apoptosis Efficacy of 5-FU on HCT116 Cells

**FIGURE 5:**
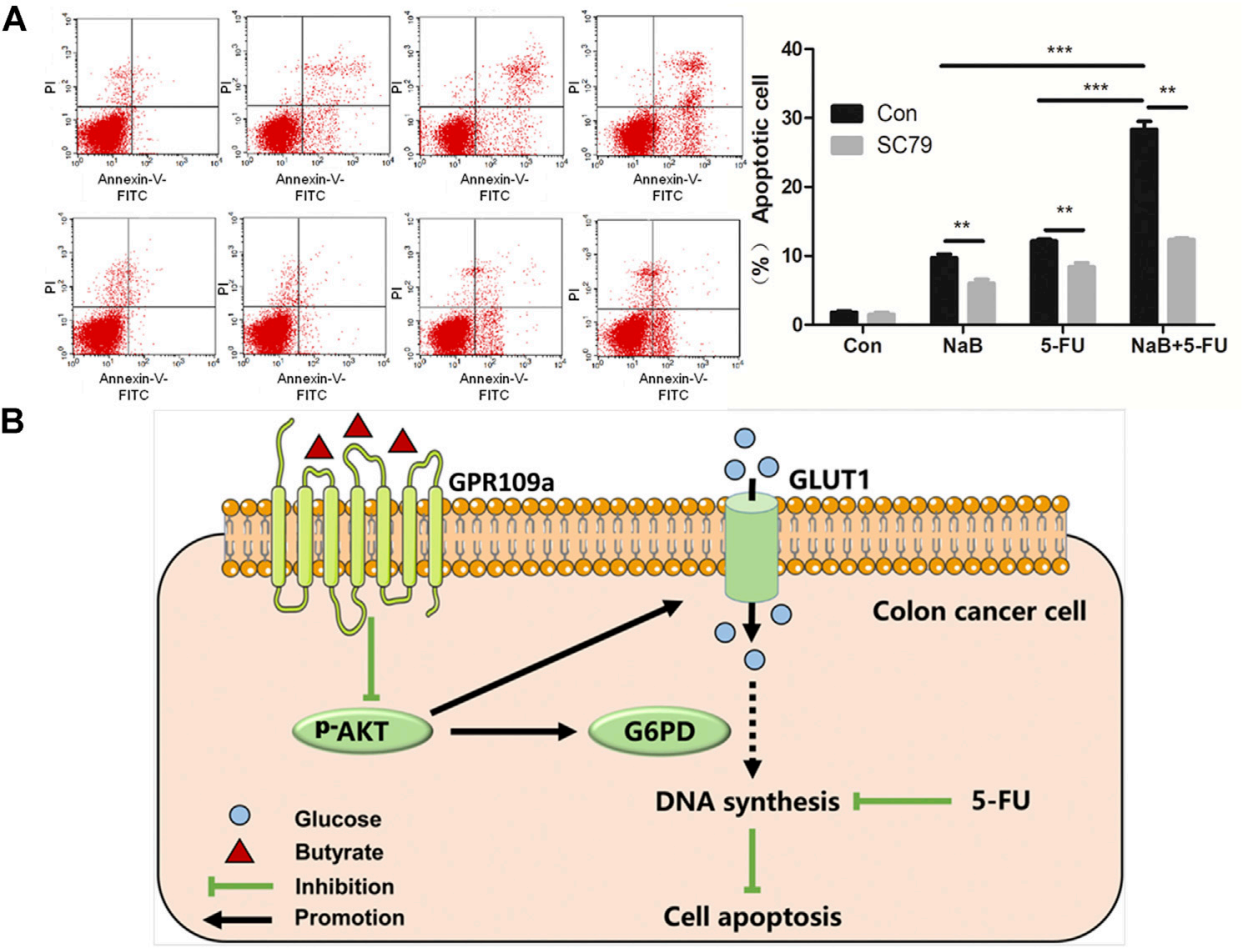
Butyrate enhanced the chemotherapeutical efficacy of 5-FU on HCT116 cells. **(A)** The apoptosis behaviors of HCT116 cells under various treating condition were measured using flow cytometry. In the first row (marked as “Con” on the left side), the apoptosis activities of HCT116 cells under four growth conditions were shown, which were treated with PBS vehicle (labeled as “Con” on the top side), 2 mM butyrate (labeled as “NaB”), 1.92 μM 5-FU (labeled as “5-FU”), or the combination of 2 mM butyrate and 1.92 μM 5-FU (labeled as “NaB+5FU”). In the second row, the apoptosis activities of HCT116 cells under various growth conditions as well as extra addition of 10.96 μM SC79 were displayed (marked as “SC79” on the left side). **(B)** A schematic view illustrating the combined effects of butyrate and 5-FU on the DNA synthesis and apoptosis of colon cancer cells.

AKT signaling pathway has been reported to be highly related to the sensitivity of colon cancer cells to chemotherapy (Radisavljevic, 2015). Therefore, it is speculated that butyrate might affect the sensitivity of colorectal cancer cells to the chemotherapeutical drug 5-FU *via* modulating the AKT signaling pathway. The apoptosis activities of HCT116 cells under various growth conditions were analyzed, which were treated with PBS vehicle, 2 mM butyrate, 1.92 μM 5-FU, or the combination of 2 mM butyrate and 1.92 μM 5-FU. In comparison to HCT116 cells added with PBS vehicle, butyrate induced apoptosis in 9% of HCT116 cells, probably resulting from its inhibitory effects on the glucose metabolism and DNA synthesis of colorectal cancer cells (Figure 5A). A high level of apoptosis of HCT116 cells was elicited by 5-FU, which was further enhanced from 13 to 22% by the combined treatment of butyrate and 5-FU, suggesting that butyrate markedly enhances the chemotherapeutical efficacy of 5-FU on colorectal cancer cells.

Extra 10.96 μM SC79 was added into the culture medium of HCT116 cells under those treating conditions, while the apoptosis effects of butyrate, and a combination of butyrate and 5-Fu were observed to be abolished by SC79, with decreased ratios of apoptotic cells from 9 to 2.5% and from 22 to 9%, respectively. These results indicate that AKT signaling pathway is essential for butyrate to enhance the sensitivity of colorectal cancer cells to the chemotherapeutical drug 5-FU.

## Conclusion and Perspectives

In summary, it is demonstrated that butyrate remarkably inhibits the glucose metabolism and DNA synthesis of colorectal cancer cells, by suppressing the abundance of membrane GLUT1 and G6PD *via* GPR109a-AKT signaling pathway (Figure 5B). Moreover, the chemotherapeutical efficacy of 5-FU on colorectal cancer cells was promoted by the combined treatment of butyrate and 5-FU, with lower DNA synthesis efficiency and higher apoptotic cell ratios.

In the global pandemic of coronavirus disease 2019 (COVID-19), clinical treatment of cancer patients is more challenging. Cancer patients including gastrointestinal cancers are more susceptible to SARS-CoV-2 infection (Liang et al., 2020), and are at remarkably higher risk of getting severe outcomes after invaded by SARS-CoV-2 (Dai et al., 2020). However, several treating approaches for tumor management showed exacerbated progress of COVID-19 disease in some circumstances. Among 105 COVID-19 patients with cancer, tumor management including immunotherapy and surgery within 40 days before the onset of COVID-19 symptoms led to enhanced frequency of critical COVID-19 outcomes (Dai et al., 2020). Treatment with immune checkpoint inhibitors (ICIs) were observed to be one predictor for severe syndromes in another cohort of 423 patients with COVID-19 and cancer (Robilotti et al., 2020). Therefore, it should be more cautious to choose appropriate therapeutical drugs for optimal management of tumors, with minimal adverse effects on SARS-CoV-2 infection. In previous studies, it is demonstrated that butyrate could improve the antivirus capacity of humans and experimental animals by maintaining the hemostasis of gut microbiota, improving the barrier functionality of mucosa, and orchestrating the innate and adaptive immune responses (Trompette et al., 2018). The inhibiting capacity of butyrate on the glucose metabolism of cancerous colonocytes as well as its beneficial effects in modulating antivirus responses reported in previous studies, make it a superior medicine or adjuvant drug in treating colorectal cancer patients in the COVID-19 pandemic.

## Data Availability

The original contributions presented in the study are included in the article/Supplementary Material, further inquiries can be directed to the corresponding author.
