# Expression of Concern: Butyrate suppresses glucose metabolism of colorectal cancer cells via GPR109a-AKT signaling pathway and enhances chemotherapy

**DOI:** 10.3389/fmolb.2026.1993113

**Published:** 2026-09-28

**Authors:** Frontiers Editorial Office

With this notice, Frontiers states its awareness of concerns regarding the image integrity of figures in “Butyrate Suppresses Glucose Metabolism of Colorectal Cancer Cells via GPR109a-AKT Signaling Pathway and Enhances Chemotherapy,” published on 29 March 2021, as raised on PubPeer: https://pubpeer.com/publications/8AAB4CD642BE71362A643D1FC3BE0F.

Frontiers conducted a post-publication investigation in accordance with our policies and the guidelines of the Committee on Publication Ethics (COPE). The authors were contacted and provided a response; however, they stated that the original raw data supporting the western blotting, flow cytometry, and metabolite measurements presented in the article are no longer available. An institutional review has been requested from the authors’ institution, Beihang University. This notice will be updated if more information comes to light.

