# Supplementary material for: Butyrate Suppresses Glucose Metabolism of Colorectal Cancer Cells via GPR109a-AKT Signaling Pathway and Enhances Chemotherapy
Source: Front Mol Biosci. 2021 Mar 29;8:634874. doi: 10.3389/fmolb.2021.634874 (PMC8039130; doi:10.3389/fmolb.2021.634874)
Supplement: Supplementary file 1 [file datasheet1.pdf]

Supplementary materials for

Butyrate suppresses glucose metabolism of colorectal cancer cells via GPR109a-AKT signaling pathway and enhances chemotherapy

Hong-Wei Geng[1], Feng-Yi Yin[1], Zhi-Fa Zhang[1], Xu Gong[1], Yun Yang[1*]

[1]Beijing Advanced Innovation Centre for Biomedical Engineering, Key Laboratory for Biomechanics and Mechanobiology of Ministry of Education, School of Biological Science and Medical Engineering, Beihang University, Beijing 100083, P. R. China

The supplementary materials includes:

Materials and Methods: Supplementary Tables 1&2

Supplementary Figures: Supplementary Figures S1 to S4

Materials and Methods:

Supplementary Table 1. siRNAs for inhibition of genes

Name	Sequence(5’-3’)
GPR109a siRNA positive-sense strand	5′-CCUUCCUGAUGGACAACUATT-3′
GPR109a siRNA antisense strand	5′-UAGUUGUCCAUCAGGAAGGTT-3′
control siRNA positive-sense strand	5′-TTCTCCGAACGTGTCACGT-3′
control siRNA antisense strand	5′-ACGTGACACGTTCGGAGAA-3′

Supplementary Table 2. Primers for quantitative RT-PCR

Name of genes	Sequence(5’-3’)	Amplicon Size (bp)
GLUT1	GGCCAAGAGTGTGCTAAAGAA<br>ACAGCGTTGATGCCAGACAG	201
GLUT2	GCTGCTCAACTAATCACCATGC<br>TGGTCCCAATTTTGAAAACCCC	183
GLUT3	GCTGGGCATCGTTGTTGGA<br>GCACTTTGTAGGATAGCAGGAAG	123
GLUT4	TGGGCGGCATGATTTCCTC<br>GCCAGGACATTGTTGACCAG	88
GLUT5	GAGGCTGACGCTTGTGCTT<br>CCACGTTGTACCCATACTGGA	77
GPR109a	ATGTTGGCTATGAACCGCCAG<br>GCTGCTGTCCGATTGGAGA	119

G6PD	CGAGGCCGTCACCAAGAAC	166
	GTAGTGGTCGATGCGGTAGA	
HK2	GAGCCACCACTCACCCTACT	249
	CCAGGCATTCGGCAATGTG	
PFK2	TTGGCGTCCCCACAAAAGT	75
	AGTTGTAGGAGCTGTACTGCTT	
ALDOA	ATGCCCTACCAATATCCAGCA	117
	GCTCCCAGTGGACTCATCTG	
PGAM1	GTGCAGAAGAGAGCGATCCG	115
	CGGTTAGACCCCCATAGTGC	
PGK1	TGGACGTTAAAGGGAAGCGG	152
	GCTCATAAGGACTACCGACTTGG	
ENO1	AAAGCTGGTGCCGTTGAGAA	217
	GGTTGTGGTAAACCTCTGCTC	
LDHA	ATGGCAACTCTAAAGGATCAGC	86
	CCAACCCCAACAACTGTAATCT	
PKM2	ATGTCGAAGCCCCATAGTGAA	118
	TGGGTGGTGAATCAATGTCCA	
PDH	TGTGAACTGAGCAGGATCTATGG	77
	GGAATGTACGATGAGGAACAACA	
PDK1	CTGTGATACGGATCAGAAACCG	191
	TCCACCAAACAATAAAGAGTGCT	
β-actin	CATGTACGTTGCTATCCAGGC	250
	CTCCTTAATGTCACGCACGAT	

Supplementary Results:

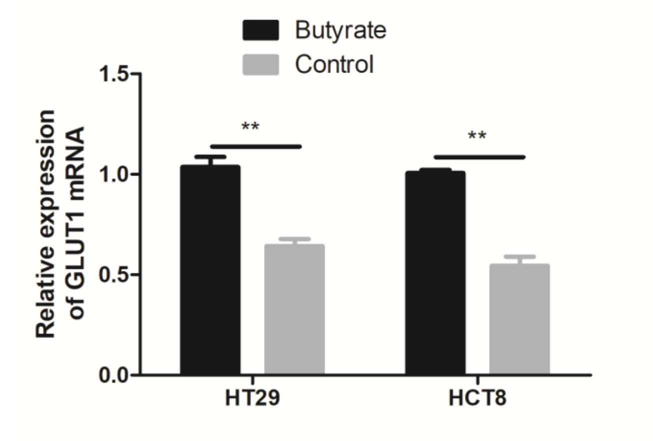

Supplementary Figure 1: Butyrate inhibits GLUT1 expression in HT29 and HCT8 cells. The mRNA level of GLUT1was tested by qPCR in HT29 and HCT8 cells after treating with 2 mM butyrate for 24 h.

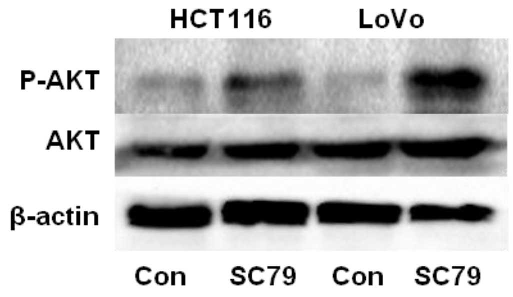

Supplementary Figure 2: SC79 promotes the expression of P-AKT in HCT116 and LoVo cells. The protein levels of total AKT and phosphorylated AKT (labeled as "P-AKT") were tested by western blotting in HT29 and HCT8 cells after incubating with 2 mM butyrate for 24 h.

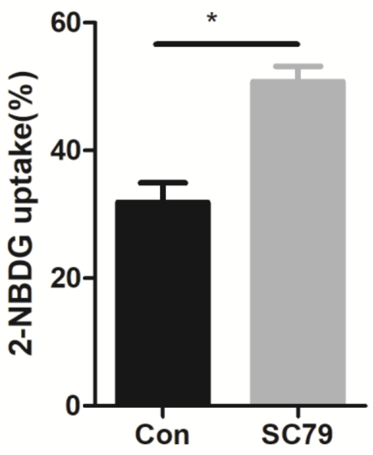

Supplementary Figure 3: SC79 promotes the uptake of 2-NBDG in HCT116 cells. The uptake of 2-NBDG was measured using flow cytometry in HCT116 cells after incubating with 10.96 μM butyrate or PBS vehicle for 24 h.

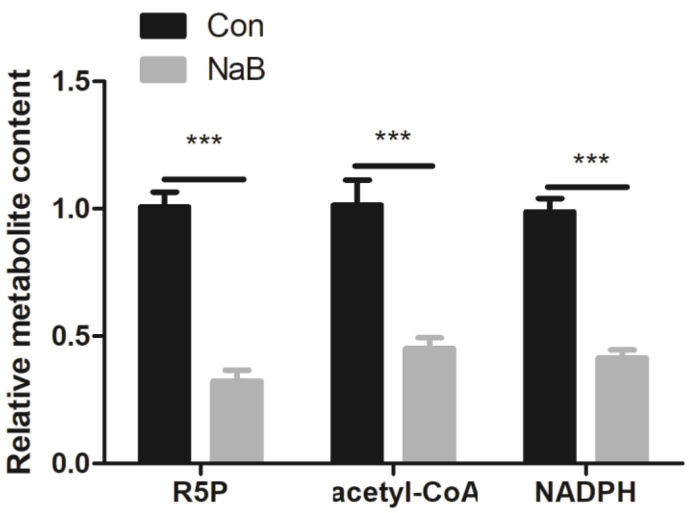

Supplementary Figure 4: Butyrate regulates the content of products related to glucose metabolism. The concentration of intracellular metabolites associated with glucose metabolism in HCT116 cells being incubated with 2 mM butyrate (labeled as “NaB”) or PBS vehicle (marked as “Con”) for 24 h were measured by liquid chromatograph-mass spectrometer/mass spectrometer (LC-MS/MS).
